# DNA Inversion Regulates Outer Membrane Vesicle Production in *Bacteroides fragilis*

**DOI:** 10.1371/journal.pone.0148887

**Published:** 2016-02-09

**Authors:** Haruyuki Nakayama-Imaohji, Katsuhiko Hirota, Hisashi Yamasaki, Saori Yoneda, Hirofumi Nariya, Motoo Suzuki, Thomas Secher, Yoichiro Miyake, Eric Oswald, Tetsuya Hayashi, Tomomi Kuwahara

**Affiliations:** 1 Department of Microbiology, Faculty of Medicine, Kagawa University, 1750–1 Miki, Kagawa 761–0793, Japan; 2 Department of Oral Microbiology, Institute of Biomedical Sciences, Tokushima University Graduate School, Tokushima 770–8503, Japan; 3 Department of Cellular and Molecular Medicine, Wakayama Medical University Graduate School of Medicine, Wakayama 641–8509, Japan; 4 Inserm UMR1043 Toulouse, France; 5 INRA USC 1360 Toulouse, France; 6 CNRS UMR5282 Toulouse, France; 7 Université de Toulouse, UPS, Centre de Physiopathologie de Toulouse Purpan (CPTP), Toulouse, France; 8 CHU Toulouse, Hôpital Purpan, Service de bactériologie-hygiène, Toulouse, France; 9 Department of Bacteriology, Faculty of Medical Sciences, Kyushu University, Fukuoka 812–8582, Japan; Centre National de la Recherche Scientifique, Aix-Marseille Université, FRANCE

## Abstract

Phase changes in *Bacteroides fragilis*, a member of the human colonic microbiota, mediate variations in a vast array of cell surface molecules, such as capsular polysaccharides and outer membrane proteins through DNA inversion. The results of the present study show that outer membrane vesicle (OMV) formation in this anaerobe is also controlled by DNA inversions at two distantly localized promoters, IVp-I and IVp-II that are associated with extracellular polysaccharide biosynthesis and the expression of outer membrane proteins. These promoter inversions are mediated by a single tyrosine recombinase encoded by BF2766 (orthologous to *tsr19* in strain NCTC9343) in *B*. *fragilis* YCH46, which is located near IVp-I. A series of BF2766 mutants were constructed in which the two promoters were locked in different configurations (IVp-I/IVp-II = ON/ON, OFF/OFF, ON/OFF or OFF/ON). ON/ON *B*. *fragilis* mutants exhibited hypervesiculating, whereas the other mutants formed only a trace amount of OMVs. The hypervesiculating ON/ON mutants showed higher resistance to treatment with bile, LL-37, and human β-defensin 2. Incubation of wild-type cells with 5% bile increased the population of cells with the ON/ON genotype. These results indicate that *B*. *fragilis* regulates the formation of OMVs through DNA inversions at two distantly related promoter regions in response to membrane stress, although the mechanism underlying the interplay between the two regions controlled by the invertible promoters remains unknown.

## Introduction

The gut is the part of the human body that is most densely populated with diverse microorganisms. The intestinal microbiota comprises diverse prokaryotic, eukaryotic and archaeal species whose populations may reach hundreds of trillions of cells. The microbial composition markedly differs throughout the intestinal tract [1], indicating that the human gut harbors unique microbe-microbe and host-microbe interactions that depend on the location in the digestive tract. Therefore, intestinal microbes must adapt to the environmental changes in their habitats including changes in nutrient availability and the levels of antimicrobial substances produced by host immunity. Because microbial adaptations reflect the characteristics of their habitats, identifying the adaptation mechanisms of gut microbes will increase the current understanding of the intestinal environment. Because the cell surface is the first point of contact with host components, cell surface adaptation is particularly important for gut bacteria to sense and survive various environmental stimuli [2].

*Bacteroides* represent a major component of the human gut microbiota [3]. Analysis of the *Bacteroides* genome revealed that these bacteria utilize a large set of dietary polysaccharides and produce many types of capsular polysaccharides on their cell surfaces [4–7]. *B*. *fragilis* can cause septicemia, appendicitis, and abdominal abscesses in humans [8]. Capsular polysaccharide A (PS A) is the most important virulence factor of *B*. *fragilis* in the induction of peritoneal abscesses [9]. PS A reportedly enhances the function of Foxp3^+^ regulatory T cells (Tregs) in the colonic lamina propria [10–12]. This finding suggests that *B*. *fragilis* produces PS A not only to evade the host immune system but also to actively modulate host physiological functions, probably contributing to microenvironmental homeostasis in the human colonic mucosa. In addition, Shen *et al*. reported that this immunomodulatory PS A is packaged into outer membrane vesicles (OMVs) and transported to host dendritic cells (DCs), resulting in the programming of DCs via a toll-like receptor 2 (TLR2) signaling pathway to induce CD4^+^, Foxp3^+^ Treg cells [13]. This study also revealed that PS A-containing OMVs protect mice from experimental colitis induced by 2,4,6-trinitrobenzene sulfonic acid (TNBS).

OMVs are produced by many Gram-negative bacteria. They participate in infection in a variety of ways, such as by delivering bacterial components (e.g., heat-labile enterotoxins) to host cells infected with enterotoxigenic *Escherichia coli* [14] and by delivering quorum-sensing signaling molecules in *Pseudomonas aeruginosa* [15]. In addition, OMVs play a role in inter-kingdom communication [16]. However, the mechanism underlying OMV formation remains unknown.

**Fig 1:**
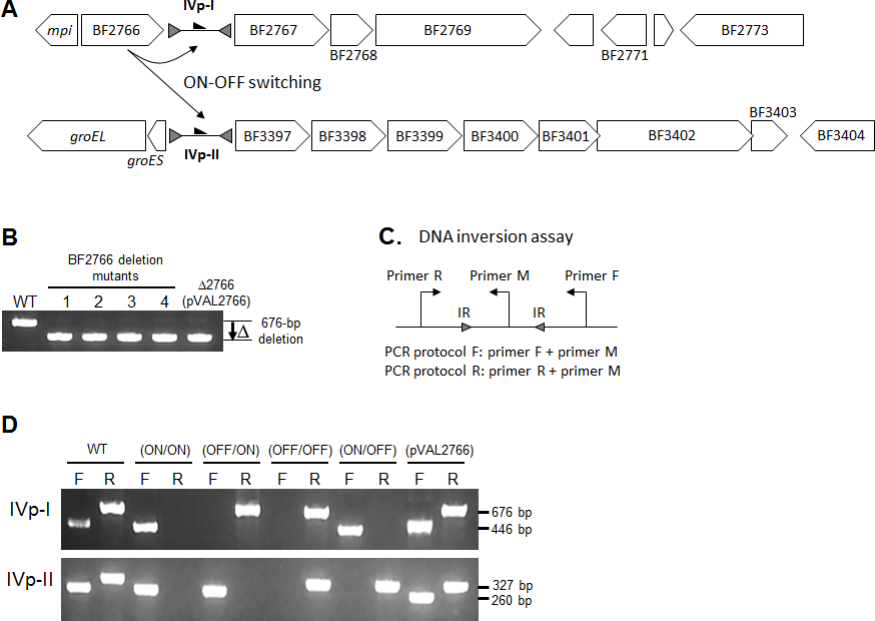
Genetic disruption of BF2766 to lock the IVp-I/IVp-II orientation. (A) An open-reading-frame map of the regions containing the BF2766-regulated invertible promoters IVp-I and IVp-II. The *mpi* gene encodes a master recombinase that controls seven PS promoter inversions. The genes downstream of IVp-I (BF2767-BF2769) are associated with extracellular polysaccharide production. IVp-II is located just upstream of the seven-gene cluster (BF3397-BF3403). The IVp-II orientation does not influence the transcription of the upstream *groES*/*EL* chaperonin genes. Detailed information about the genes shown in this panel is summarized in S1 Table. Gray arrowheads: inverted repeat sequences (IRs). Black arrowheads: promoter orientation. (B) PCR-based confirmation of a 676-bp internal deletion of BF2766. The four mutants with different genotypic combinations of IVp-I/IVp-II orientation were used for the following analyses. (C) Schematic of the primer design to detect IVp-I/IVp-II orientations. (D) BF2766 mutants showing the different IVp-I/IVp-II-locked patterns. F and R indicate the primer pair shown in panel C. In a wild-type sample, PCRs with both the F and R primers produced bands of the expected size (indicated on the right side), showing that IVp-I/IVp-II promoter inversion occurs *in vitro*. The locked patterns of IVp-I/IVp-II in the BF2766 mutants are indicated above the lane. For example, ON/ON indicates that both promoters are locked ON relative to downstream genes. Plasmid complementation of the mutants with intact BF2766 restored IVp-I/IVp-II promoter inversion. The IVp-I OFF genotype was prevalent in the wild type, but plasmid complementation abrogated this deviation.

*B*. *fragilis* regulates the expression of PS A and six other capsular polysaccharides via phase-variable promoter inversions [17]. *B*. *fragilis* DNA inversions have also been associated with variable expression of outer membrane proteins in the SusC/SusD family [18], surface proteins associated with the autoaggregative phenotype [19], and high-molecular-weight extracellular polysaccharide (EPS) [20]. These phase variations are probably associated with the modulation of host immunity, nutrient uptake, and biofilm formation, respectively. Three master DNA invertases that globally control promoter inversions at many distant regions have been identified in *B*. *fragilis*: Mpi inverts 13 regions, seven of which are PS promoters [21]; Tsr0667 is responsible for shufflon-type multiple DNA inversions at three SusC/SusD outer membrane gene clusters and nine other invertible regions [18]; and Tsr19 (corresponding to the BF2766-encoded DNA invertase in *B*. *fragilis* YCH46) inverts the local and distant promoters (Fig 1A) that confer the large-capsule phenotype on this species [20, 22]. The BF2766-regulated invertible regions are unique in that the local promoter (designated as IVp-I in Fig 1A) shifted in the OFF orientation relative to the three downstream EPS biosynthesis genes in cultured media and in a majority of tested human feces samples [20], which indicates that continuous expression of this EPS is not essential for colon colonization by *B*. *fragilis*.

In the present study, the two BF2766-regulated invertible promoters of *B*. *fragilis* YCH46 were further analyzed to determine the role of their associated proteins in surface structure modification. The results indicated that OMV formation in *B*. *fragilis* is attributed to the two BF2766-regulated regions. Vesiculating *B*. *fragilis* cells exhibited increased resistance to bile and certain types of human defensins. The data presented here may enhance the current understanding of the genetic basis of OMV formation and the role (s) of these vesicles in mutualistic colonization by *B*. *fragilis*.

## Results

### The BF2766-encoded DNA invertase mediates local and distant promoter inversions

The *B*. *fragilis* genome contains at least eight capsular polysaccharide biosynthesis loci (PS loci). Among these, seven include invertible promoters, which enable the bacteria to alter the surface glycan in a phase-variable manner [17]. These PS promoter inversions are mediated by a single serine recombinase, Mpi. The *B*. *fragilis* gene that encodes Mpi co-localizes with a tyrosine recombinase gene (BF2766 in strain YCH46, which corresponds to *tsr19* in strain NCTC9343) in a head-to-head orientation (Fig 1A). The BF2766 gene reportedly regulates promoter inversions in two regions: the immediate downstream promoter of an extracellular polysaccharide (EPS) operon and a gene cluster that includes a lipoprotein gene [20, 22]. Detailed information about the genetic composition of these regions is summarized in S1 Table. In the present study, these two invertible promoters are designated IVp-I and IVp-II, respectively.

The IVp-I inversion has been associated with high-molecular-weight EPS production in *B*. *fragilis* NCTC9343, but the function of IVp-II remains uncharacterized [20]. To further characterize the IVp-I/IVp-II-related *B*. *fragilis* phenotype, BF2766, which encodes the master recombinase for these promoters, was deleted in strain YCH46 (Fig 1B). The BF2766 deletion abrogated DNA inversions at both IVp-I and IVp-II. Among the 96 mutant colonies screened, we obtained only one genotype, in which the IVp-I/IVp-II promoters were locked in the OFF/ON orientation (IVp-I being OFF and IVp-II being ON) relative to downstream genes, indicating that the OFF/ON genotype was predominant in laboratory broth culture.

To determine the effect of IVp-I/IVp-II promoter inversions on *B*. *fragilis* physiology, four locked mutants (IVp-I/IVp-II locked in the ON/ON, ON/OFF, OFF/ON or OFF/OFF orientation relative to downstream genes) were constructed. These four mutants were obtained by complementing the OFF/ON mutant with a plasmid harboring BF2766 and then curing the plasmid in nonselective medium (Fig 1D). The PCR-based determination of the resultant orientations showed that the IVp-I-ON population (indicated by “F”) was smaller than the IVp-I-OFF population (indicated by “R”) in wild-type cells. No differences were observed in the frequencies of the IVp-II orientation. The deviations in the IVp-I orientation disappeared when the BF2766 mutant was complemented with a plasmid encoding BF2766, which suggests that BF2766 expression is suppressed in wild-type cells or that a regulatory mechanism controls IVp-I orientation.

It has been reported in NCTC9343 that the number of inverted repeat sequences (IRs) between the promoter and the downstream genes is inversely correlated with the transcriptional levels of the EPS biosynthesis operon downstream of IVp-I [20]. Consequently, the *tsr19* (ortholog of BF2766) mutant with a single IR between the promoter and the downstream genes showed increased EPS production, and these bacteria did not pelleted after centrifugation, whereas mutants with two or three IRs generated relatively compact pellets. Both of the locked IVp-I ON BF2766 mutants generated in the present study have two IRs between the promoter and downstream gene (S1 Fig).

### Cell surface morphology of the four types of IVp-I/IVp-II-locked mutants

**Fig 2:**
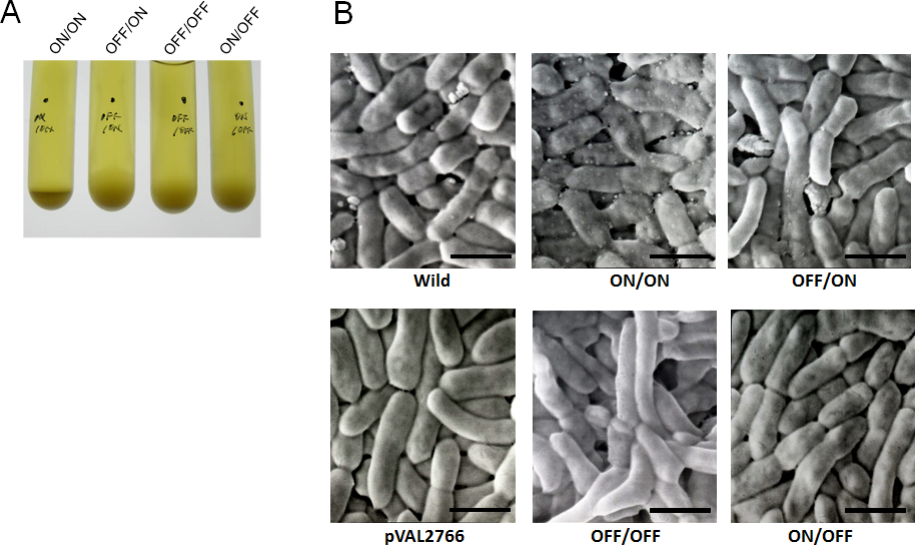
Orientation-specific phenotypes of BF2766 mutant *B*. *fragilis* cells with respect to IVp-I/IVp-II. (A) The four types of IVp-I/IVp-II-locked mutants were anaerobically cultured in GAM broth overnight. Each culture was mixed to homogeneity and grown in an anaerobic chamber. Periodically, cell sedimentation was visually compared. ON/ON cells sank to the bottom faster than the other three genotypes, generating a more compact cell pellet. The promoter patterns are indicated above the tubes. (B) Scanning electron micrographs of wild-type and mutant cells. Plasmid-complemented BF2766 mutant cells (pVAL2766) were also examined. The ON/ON-locked mutant produced a large number of vesicle structures on the cell surface. Bar: 2 μm.

**Fig 3:**
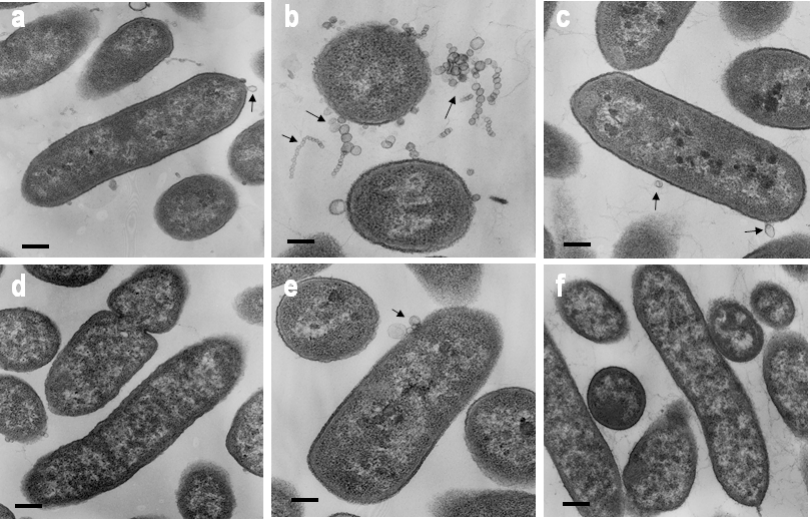
Transmission electron micrograph of BF2766 mutant *B*. *fragilis* cells. *B*. *fragilis* cells were fixed with 2% glutaraldehyde/PBS and analyzed by TEM to observe OMVs. OMVs (arrows) were observed in wild-type *B*. *fragilis* (panel a). The ON/ON locked mutant (panel b) produced a large number of OMVs that formed clusters or chains. Plasmid complementation of the ON/ON mutant with intact BF2766 decreased OMV production to wild-type levels (panel d). The level of OMV production was similar among the OFF/ON (panel c), OFF/OFF (panel e), and ON/OFF (panel f) mutants. Bar: 0.1 μm.

As shown in Fig 2A, ON/ON mutant cells sedimented more rapidly than other IVp-I/IVp-II- locked mutants. This rapid cell sedimentation rate indicated that the ON/ON mutant has a unique phenotype that is associated with autoaggregation. Scanning electron microscopy (SEM) showed that the cell surface of the ON/ON mutant was markedly different from that of the other locked mutants and that the ON/ON cells displayed a large number of surface vesicle structures (Fig 2B). Transmission electron microscopy (TEM) also demonstrated that the ON/ON mutant produces a large number of 20-50-nm OMVs whereas OMVs were observed in only a small number of wild-type and other mutant cells (Fig 3). The plasmid complementation of ON/ON mutant with BF2766 decreased OMV production. Together, these results suggested that the cooperative action of IVp-I and IVp-II regulates the gene products involved in OMV formation in *B*. *fragilis*.

To examine whether the hypervesiculation phenotype was strain-specific, we engineered the ON/ON genotype in NCTC9343. SEM analysis demonstrated that hypervesiculation was also observed in ON/ON genotypes derived from NCTC9343 (S2 Fig).

### Role of IVp-I/IVp-II on downstream gene expressions

**Fig 4:**
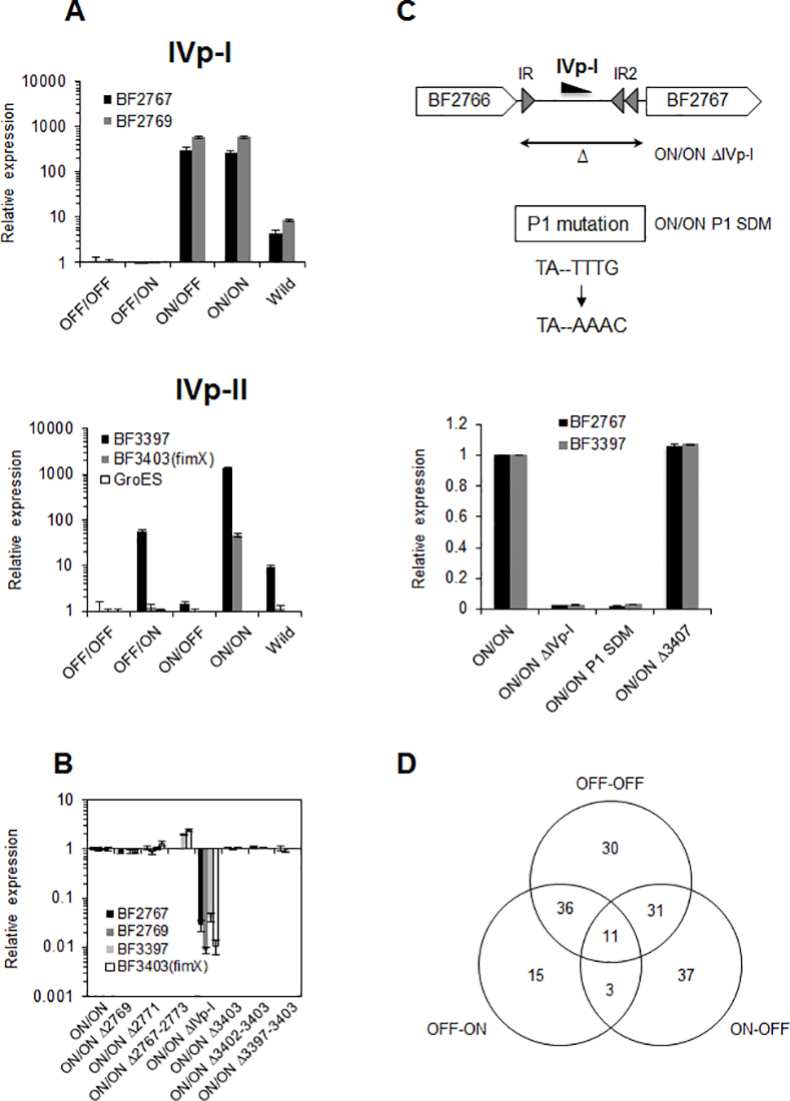
Orientation-specific unique transcriptional regulation by invertible IVp-I/IVp-II. (A) The expression of EPS biosynthesis genes (represented as BF2767 and BF2769) and a gene cluster (represented as BF3397 and BF3403) were dependent on IVp-I and IVp-II, respectively. The transcriptional level of the IVp-II-regulated gene cluster in the hypervesiculating mutant (ON/ON) was increased 25- to 40-fold over that of the OFF/ON mutant. (B) IVp-I was required for the full expression of the gene cluster downstream of IVp-II. A variety of deletions were generated within the EPS production locus or at the IVp-II-regulated gene cluster in the hypervesiculating mutant (ON/ON). Only the deletion of the IVp-I-containing invertible DNA region affected the expression of IVp-II-regulated genes. (C) The effects of IVp-I activity and IVp-I-related IRs on IVp-II activity. IVp-I activity was attenuated after replacing the -7 sequence (TAnnTTTG is the *Bacteroides* consensus promoter sequence at -7) with an inactive form (TAnnAAC) by using site-directed mutagenesis. Two IRs were present between IVp-I and BF2767 in the ON/ON mutant obtained in this study. These IRs and the entire invertible fragment containing IVp-I were deleted to examine the role of these elements in IVp-II activity. (D) Venn diagram comparing differentially expressed genes in *B*. *fragilis* mutants with different IVp-I/IVp-II orientations. The number of differentially expressed genes (> 4-fold change) in each genotype was compared with the ON/ON genotype. The expression levels of 11 genes were specifically elevated in the ON/ON mutant (S2 Table).

A previous study based on the *xylE* reporter assay showed that IVp-II has no effect on downstream gene expression [20]. However, the data obtained in the present study indicated that IVp-II promoter orientation is associated with OMV production. Thus, qPCR analysis of the IVp-I and IVp-II downstream genes was performed on the four locked mutants. As shown in Fig 4A, gene expression in both regions depended on the orientation of IVp-I and IVp-II. The expression of BF2767 and BF2769 (the first and last genes of the EPS production operon) was induced only when IVp-I was ON relative to these genes. The transcriptional levels of BF2767 and BF2769 in the parental strain were lower than in the IVp-I-ON mutants, consistently with the results of the PCR analysis (Fig 1D), in which the population of IVp-I-ON genotypes was low in *in vitro* growth media. Similarly, IVp-II was necessary for the transcription of BF3397 (the gene immediately downstream of IVp-II). As reported [22], the IVp-II promoter did not have a role in the expression of the upstream *groES*/*EL* operon.

Interestingly, the expression of the genes downstream of IVp-II was much higher in ON/ON cells than in OFF/ON cells (with 25- and 40-fold increases in BF3397 and BF3403 expression, respectively). This effect was not observed for BF2767 and BF2769, for which there was no observed difference between the ON/ON and ON/OFF genotypes. Therefore, the IVp-I-regulated genomic region might have a regulatory role in IVp-II-regulated gene expression. To examine this hypothesis, we deleted a region, beginning with the genes immediately downstream of IVp-I (BF2767) and extending to just upstream of the *upxY* genes (BF2774) in the capsular polysaccharide biosynthesis locus (PS-7), in the ON/ON mutant (Fig 1A). However, these deletions had no effect on the expression levels of IVp-II-regulated genes (Fig 4B), the expression levels of BF3397 and BF3403 remained similar to those detected in the original ON/ON mutant.

The IVp-I promoter region was subsequently deleted from the ON/ON mutant. This deletion reduced the expression of IVp-II-regulated genes to levels similar to those in the OFF/ON genotype (Fig 4B). Bayley *et al*. reported that an alteration of the *Bacteroides* promoter sequence at -7 (TAnnTTTG) to TAnnAAAC resulted in the loss of promoter activity [23]. When this same alteration was introduced into IVp-I through site-directed mutagenesis, the enhancement effect on IVp-II activity was abolished (Fig 4C). These results suggest that IVp-I activity is necessary for the overexpression of IVp-II-regulated genes.

DNA microarray analysis was performed with the four locked mutants to identify genetic regions whose alterations in expression were specifically associated with the ON/ON mutant. Only 11 genes were differentially expressed (>4-fold change) in the ON/ON mutant (Fig 4D). As shown in S2 Table, all of the genes were upregulated. Notably, ON/ON-specific expression was limited to the IVp-II-related region (BF3397-BF3403, BF3406-BF3407 and BF3410), with the exception of BF4531. As shown in Fig 4C, genetic disruption of BF3407 (encoding an extracytoplasmic function (ECF) sigma factor) in the ON/ON background did not change the expression of BF3397 (the gene immediately downstream of IVp-II). These results indicate that the genetic region that is responsible for the hypervesiculation phenotype observed in ON/ON mutants is restricted to IVp-I and IVp-II-regulated loci.

### Both EPS and IVp-II-regulated gene products are required for OMV production

Deletions of IVp-I-related genes were then examined in the ON/ON background. Deletion of the EPS production gene, BF2769 (encoding a tyrosine protein kinase) resulted in drastically reduced OMV production (S3 Fig). Plasmid complementation of this mutant with intact BF2769 restored OMV production to a level similar to that observed in the parental ON/ON mutant. In contrast, OMV production was not affected by the deletion of BF2771, which is located outside the EPS production operon.

**Fig 5:**
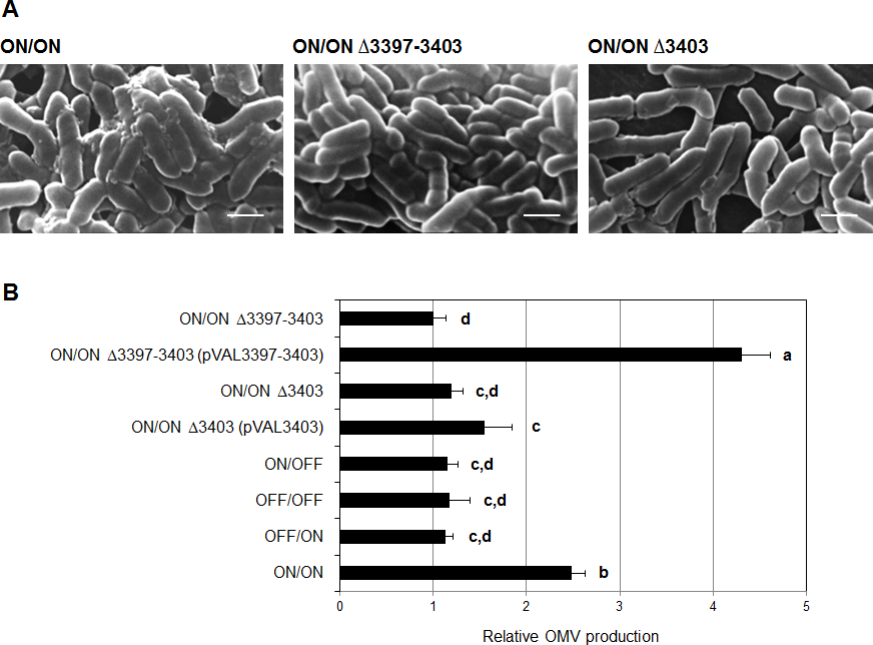
The role of IVp-II-regulated genes in *B*. *fragilis* OMV formation. (A) SEM was used to compare OMV formation. The IVp-II-regulated gene cluster was entirely (Δ3397–3403) or partially (only BF3403, the last gene of the cluster) deleted in the ON/ON genetic background of *B*. *fragilis* YCH46. Bar: 1 μm. (B) Comparison of OMV production. OMV production was quantified by measuring the protein content of culture supernatants. The graph indicates OMV production relative to that of the ON/ON (Δ3397–3403) mutant. The data are expressed as means ± standard deviation. The bars labeled with different letters indicate significant differences at *p* < 0.05.

Next, the roles of IVp-II-regulated genes in OMV production were examined. As shown in Fig 5A, deletion of all the IVp-II-regulated genes (BF3397-BF3403) in the ON/ON background abrogated vesiculation, and almost all of the cells exhibited a smooth cell surface. Plasmid complementation of this mutant with a fragment containing BF3397-BF3403 resulted in an approximately two fold increase in OMVs over that of the parental ON/ON mutant when OMV production was quantified according to the protein content in culture supernatants (Fig 5B).

Together, these data indicate that the high OMV production in *B*. *fragilis* is induced by the cooperative action of gene products encoded by the IVp-I- and IVp-II-regulated regions and that the overexpression of IVp-II-regulated genes is an essential part of the process.

### Proteomic analysis of OMVs

**Fig 6:**
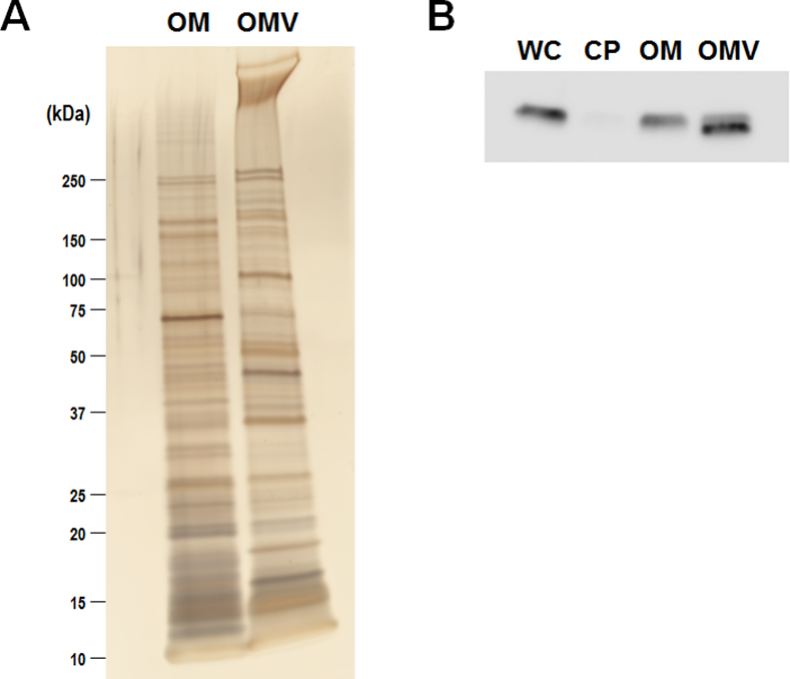
Proteomic analysis of OMVs purified from ON/ON mutant *B*. *fragilis* cells. (A) SDS-PAGE patterns of outer membrane (OM) and OMV fractions. (B) Localization of FLAGtag-fused BF3397 protein. One micrograms of the each purified fraction was separated by SDS-PAGE and BF3397 localization was determined by Western blotting with anti-FLAGtaq antibody. WC, whole cell; CP, cytoplasmic fraction; OM, outer membrane fraction; and OMV, outer membrane vesicle fraction.

The proteins in outer membrane and OMV fractions of ON/ON mutant of *B*. *fragilis* YCH46 were prepared as described by Elhemawy et al. [24] and separated by SDS-PAGE (Fig 6A). The protein profiles in OMVs were different from those in outer membrane, indicating the mechanisms for sorting and exclusion of proteins into OMV must exist in *B*. *fragilis*.

The ON/ON mutant derivative, in which BF3397 protein was engineered to be produced as C-terminal FLAG tag fusion protein, was separated into whole cell, cytoplasmic, outer membrane and OMV fractions. The proteins in each fraction were separated by SDS-PAGE, transfer to PVDF membrane, and then blotted with anti-FLAG antibody. As shown in Fig 6B, BF3397 was localized in outer membrane and OMV fractions. Interestingly, the size of BF3397 was different in outer membrane and OMV fractions. BF3397 in OMV fraction was smaller than that in outer membrane fraction. Because FLAGtag was fused to C-terminal end, BF3397 in OMVs was predicted to be N-terminally processed. This processed form of BF3397 was enriched in the OMVs.

### Physiological roles of OMV production in *B*. *fragilis*

**Fig 7:**
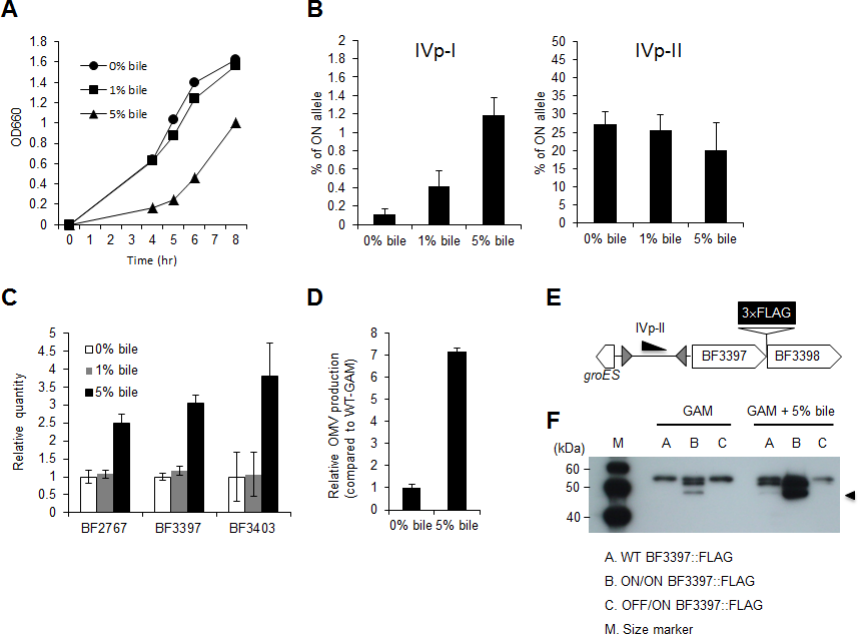
Effect of bile exposure on *B*. *fragilis* OMV formation. (A) Growth of *B*. *fragilis* YCH46 in GAM supplemented with the indicated bile concentrations. (B) qPCR was used to determine the relative abundance of the IVp-I-ON or IVp-II-ON genotypes after exposure to the indicated bile concentration. (C) Effect of bile exposure on the expression of an IVp-I-regulated EPS production gene (BF2767) and IVp-II-regulated membrane protein genes (BF3397 and BF3403). (D) OMV production of *B*. *fragilis* increased 7-fold after exposure to 5% bile. (E) Schematic of the 3 x FLAG-tag fusion to the C-terminus of the BF3397-encoded protein. (F) Enhancement of the BF3397-encoded membrane protein in response to 5% bile. A *B*. *fragilis* YCH46 derivative with a 3 x FLAG-tag sequence fused to BF3397, as shown in panel E, was exposed to 5% bile: the production of BF3397 was assessed by Western blotting with an anti-FLAG antibody. Upper, middle, and lower bands indicate the anti-FLAG tag antibody used for immunoprecipitation, BF3397 (unprocessed form), and N-terminally processed BF3397 (indicated by arrowheads), respectively.

Gram-negative bacteria produce OMVs in response to membrane stress. Pumbwe *et al*. reported that *B*. *fragilis* increased its OMV production when exposed to bile, indicating that OMV formation is an adaptation for bile resistance [25]. Therefore, we monitored the population levels of cells with IVp-I-ON or IVp-II-ON genotypes by using orientation-specific qPCR in culture media with or without bile acid. Because the physiological bile concentration in the human gut ranges from 0.1% to 1.3% [26, 27], *B*. *fragilis* YCH46 cells were treated with 1.0% or 5.0% bile (final concentration). *B*. *fragilis* YCH46 showed growth in medium containing 1% bile that was comparable to its growth in bile-free medium (Fig 7A). Even in the presence of 5% bile, *B*. *fragilis* grew to an OD_600_ of nearly 1.0 within 8 h, although the lag phase was apparently prolonged. Analysis by qPCR with DNA extracted from mid-log-phase cultures (5–6 h point in Fig 7A) with and without bile showed that exposure to bile increased the number of cells with the IVp-I-ON genotype in a dose-dependent manner (3.78- and 10.8-fold increase with 1.0% and 5.0% bile exposure, respectively), whereas no significant effect was observed on the population levels of cells with an IVp-II-ON genotype (Fig 7B). In accord with these findings, the expression levels of BF2767 (IVp-I-regulated) and BF3397 and BF3403 (both IVp-II-regulated) were elevated when the cells were exposed to 5.0% bile (Fig 7C). Furthermore, OMV production based on the protein concentration in culture supernatants was increased 7-fold after exposure to 5.0% bile than in cells without bile exposure (Fig 7D).

To further confirm the response of IVp-I- and IVp-II-regulated genes to bile, we monitored the intracellular level of BF3397 by using a recombinant protein fused to a C-terminal FLAG tag (Fig 7E). Following cultivation with or without 5% bile, the level of the BF3397-encoded protein was quantified by Western blotting with an anti-FLAG antibody. As shown in Fig 7F, the level of the BF3397-encoded membrane protein in wild-type cells increased in response to bile exposure, whereas no increase was observed in the OFF/ON mutant. As shown in Fig 6, N-terminally processed BF3397 (indicated by arrowhead), which was specifically localized in OMVs, was observed in wild type cells exposed to bile as well as in ON/ON mutant. Interestingly, the protein level was also increased in the ON/ON-locked mutant, suggesting that bile exposure induces another regulatory mechanism for BF3397 protein production.

### Resistance to antimicrobial substances

**Fig 8:**
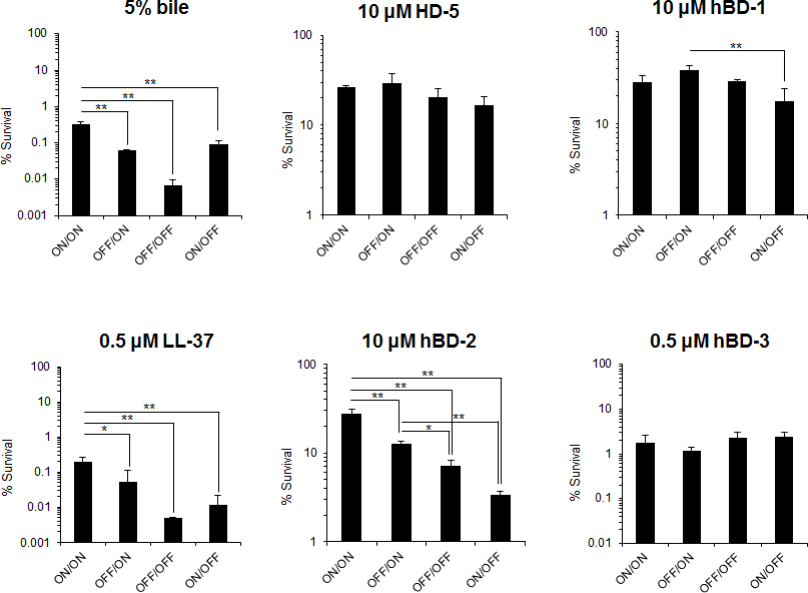
Sensitivity of *B*. *fragilis* locked IVp-I/IVp-II-locked mutants to antimicrobial substances. Mid-log growth-phase mutant cells (ON/ON, OFF/ON, OFF/OFF, or ON/OFF) were suspended in PBS (10^7^ cfu/ml) and mixed with bile, LL-37 or defensins (human α-defensin 5 [HD-5], or human β-defensins-1 [HBD-1], -2 [HBD-2], or-3 [HBD-3]) at the indicated concentrations. After the mixtures were incubated at 37°C for 30 min (for bile) or 1 h (for LL-37 and defensins), the surviving cells were enumerated. The columns show the ratios of surviving cells to initial viable cell numbers. The data are expressed as the mean ± standard deviation. The differences were statistically analyzed by ANOVA, followed by Tukey’s test. *P*-values of less than 0.05 and 0.01 are indicated as * and **, respectively.

As described above, bile exposure increased the population level of the ON/ON genotype, which led to the overexpression of IVp-II-regulated genes, and thus in a hypervesiculation phenotype. To examine the role of OMV production in adaptation to cell envelope stress, we compared the survival of the four *B*. *fragilis* locked IVp-I/IVp-II mutants following contact with antimicrobial compounds (bile, LL-37 and human defensins). The results indicated that the ON/ON genotype was more resistant to bile, LL37 and certain types of human defensins than the other three genotypes (Fig 8). Interestingly, we observed selective resistance against human defensins. Among the defensins tested (human α-defensin 5 and human β-defensins 1, 2, and 3), a difference was observed only with human β-defensin 2, to which the ON/ON genotype was most resistant. The survival rates after contact with the other defensins were similar among the four locked mutants.

To test the protective effect of OMVs from bile, OMVs were purified from ON/ON mutant culture. The purified OMVs were added to indicated concentration into the cell suspensions of non-vesiculating mutant (OFF/ON or OFF/OFF), and the cells were exposed to 5% bile (S4 Fig). The survival rate of the cells was increased the OMV concentration was increased. The survival rate of the cells with 50 μg/ml OMVs was significantly higher than the cells without OMVs (*p*<0.01).

### Genotypic prevalence of IVp-I/IVp-II *in vivo*

**Fig 9:**
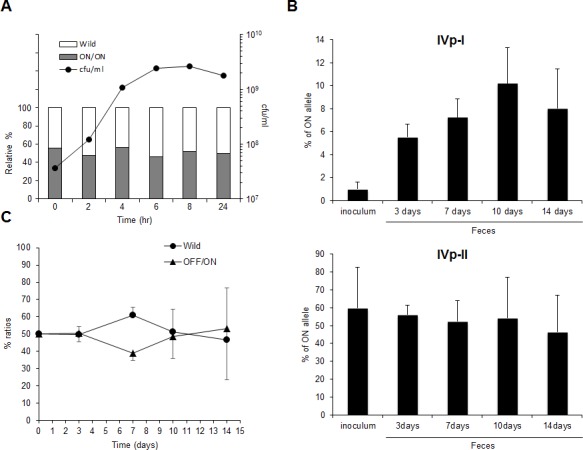
Comparative analysis of the in vitro and in vivo growth of IVp-I/IVp-II-locked mutants. (A) Competitive growth assay of ON/ON *B*. *fragilis* mutant and its parental YCH46 stain *in vitro*. Equivalent cell mixture of wild and mutant *B*. *fragilis* cells was inoculated into GAM broth. Closed circles indicate the periodical change of total viable cell number in GAM broth. Columns indicate the relative abundance of the wild and mutant *B*. *fragilis* cell in GAM broth. (B) IVp-I/IVp-II ON/OFF prevalence during *in vivo* gut colonization IVp-I/IVp-II under *in vivo* gut colonization by *B*. *fragilis*. A cell suspension (7.0 x10^7^ CFU in total) of wild-type *B*. *fragilis* was inoculated into three 8-week-old male BALB/cA germ-free mice via gavage. At 3, 7, 10, and 14 days after inoculation, feces samples were collected. DNA was extracted from the initial inoculum and the collected feces samples, and the ON/OFF ratio of IVp-I/IVp-II in each sample was determined by qPCR. The data are expressed as means ± standard deviation. The bars labeled with different letters indicate significant differences at *p* < 0.05. (C) Competitive growth assay of IVp-I/Vp-II-locked *B*. *fragilis* mutant and its parental YCH46 stain. Equivalent cell mixture of wild and the OFF/ON mutant cells was inoculated into BALB/c germ-free mice. Colony PCR was performed on at least 96 colonies per sample with primer pair encompassing the deletion site in BF2766 to compare population levels of mutants (closed triangles) with wild type (closed circles) cells in the mouse intestine. In this experiment, mice were kept in a vinyl isolator to maintain their gnotobiotic conditions.

As shown in Fig 9A, *in vitro* growth of hypervesilating cells was equivalent to that of wild type *B*. *fragilis*. The *B*. *fragilis* YCH46 wild-type strain was inoculated into three 8-week-old male BALB/cA mice by gavage, and the fecal population levels of cells with IVp-I-ON or IVp-II-ON genotypes were monitored by qPCR at 3, 7, 10, and 14 days after inoculation (Fig 9B). In the cell suspension used for inoculation, the IVp-I-ON population was only 1.03±0.01%, whereas the population of IVp-II-ON cells was 59.72±0.23%. After gut colonization, the fecal IVp-I-ON population periodically increased, whereas IVp-II-ON population level remained stable during the experimental period. This trend was similar to that for bile exposure shown in Fig 7B, indicating that IVp-I/IVp-II-regulated OMV production plays a role in *B*. *fragilis* adaptation during gut colonization. However, the gut colonization until 14 days after inoculation was not different among wild type and OFF/ON mutant strains (Fig 9C), indicating hypervesiculation is one of the adaptation phenotypes under special condition.

## Discussion

Some pathogenic bacteria have evolved phase-variation systems for cell surface structures to evade host immunity (e.g., the flagella of *Salmonella* Typhimurium and the pili of *Neisseria meningitidis*) [28–30]. Recent genomic analysis has revealed that phase-variation systems are abundant among *Bacteroides* species, which are dominant members of the human gut microbiota. *Bacteroides* generate population heterogeneity among capsular polysaccharides [17, 21] or in the SusC/SucD family of outer membrane proteins [18] by various mechanisms of DNA inversion (simple promoter inversion or multiple shufflon-type DNA inversion). The results of the present study included the novel observation that promoter inversion is a mechanism for OMV formation in *B*. *fragilis*.

Because enhanced OMV production was observed only in the ON/ON genotype with respect to IVp-I/IVp-II, and because IVp-II-regulated gene expression was specifically increased in this mutant, an elevated outer membrane protein level driven by this gene cluster was considered necessary for hypervesiculation. IVp-I was essential for the expression of the downstream EPS biosynthesis genes, which confers a large capsule (LC) phenotype to *B*. *fragilis* cells [20]. Patrick *et al*. initially described the LC phenotype in a population of cells surrounded by a thick halo (recently recognized as EPS) after negative staining [31]. The EPS production was reported to markedly increase in IVp-I-ON mutant with a single IR between IVp-I and the first EPS gene [20]. The ON/ON mutants derived from YCH46 and NCTC9343 obtained in this study have two and one IR between IVp-I and the first EPS gene, respectively (S1 Fig). Because the ON/OFF genotype reduced OMV formation when compared with the ON/ON genotype in both *B*. *fragilis* strain (Fig 3 and S2 Fig), EPS production is essential for OMV formation but EPS overproduction may not be necessary for hypervesiculation.

Despite the variety of physiological and pathological roles of OMVs in Gram-negative bacteria [32, 33], the mechanism of OMV formation has remained a mystery. However, several models of OMV formation have been proposed [33]: (i) decreased in lipoprotein linkage between the outer membrane and peptidoglycan, (ii) increased membrane turgor pressure reflecting the periplasmic accumulation of peptidoglycan components or misfolded proteins, and (iii) charge repulsion resulting from a modified form of lipopolysaccharides. Similar to the first (i) OMV-forming mechanism described above, a hypervesiculation phenotype was observed in *Vibrio cholerae* when the expression of the gene for the major outer membrane protein, OmpA, was repressed through *vrrA*, a non-coding RNA [34]. In contrast, a study on *Salmonella* Typhimurium reported the enrichment of an 18-kDa outer membrane protein, PagC, in OMV: the overexpression of this protein provoked OMV formation [35]. Because PagC is induced in acidified macrophage phagosomes [36], PagC-mediated OMVs might contain molecules that modify the phagosomal environment making this habitat suitable for *Salmonella*. These results indicate that some pathogens possess machinery to actively produce OMVs that modify the surrounding environment.

The mode of *B*. *fragilis* OMV formation demonstrated herein is similar to that of *Salmonella*, because OMV formation is induced by overproduction of outer membrane proteins and by environmental stress. The proteomic analysis revealed that the IVp-II-regulated outer membrane protein, BF3397, localizes in both outer membrane and OMVs. It is noteworthy that BF3397 protein was localized in OMV fraction as N-terminally processed form, which clearly differed from that of outer membrane in size. It is important to know how BF3397 protein is processed to elucidate the pinching off mechanism of OMV from outer membrane.

Pumbwe *et al*. reported that *B*. *fragilis* OMV formation is promoted by bile exposure [25]. In accord with those data, the results of the present study demonstrated that a hypervesiculating *B*. *fragilis* strain (ON/ON genotype) showed the most resistance to bile among the four locked genotypes. Quantitative PCR showed that the IVp-I-ON population was increased when *B*. *fragilis* was exposed to bile, whereas the ON/OFF ratio of IVp-II was not changed. The FLAG-tag fused BF3397 protein level (both unprocessed and processed forms) was elevated after bile exposure reflecting the enhancement of IVp-II activity through an increase in the IVp-I-ON population (Fig 7F). The hypervesiculating strain was not only resistant to bile, but it also showed greater resistance to certain types of human defensins. Defensins are cationic antimicrobial peptides (CAMPS) that are produced by various cells as a component of innate immunity. α-defensin 5 (HD5) and 6 (HD6) are the most common defensin molecules expressed at the base of the crypts of Lieberkuhn [37]. Colonic epithelial cells constitutively produce human β-defensin (HBD1). The expression of HBD-2, -3, and -4 is induced by various inflammatory and bacterial stimuli [38].

As shown in Fig 8, the ON/ON genotype specifically showed resistance to HBD-2, whereas its resistance to other human defensins (HD-5, and HBD-1, 3) was similar to that of the other three genotypes. This result is interesting because HBD-2 is induced in the gut under inflammatory conditions, such as in Crohn’s disease and ulcerative colitis [38]. Notably, no difference in the cell survival was observed with HD-5 and HBD-1. Therefore, OMV production via the coordinated expression of IVp-I/IVp-II-regulated genes might be one of the defense mechanisms *B*. *fragilis* uses to escape membrane stressors in the inflamed gut. This idea well explains the observation that population of ON/ON genotype is very low *in vitro* culture or in feces from previously reported healthy volunteers: six of seven tested was IVp-I was OFF orientation [20]. These findings raised the idea that *B*. *fragilis* IVp-I-ON population level might be a good indicator for lower gut inflammatory condition. Animal experiments demonstrated that the fraction of *B*. *fragilis* cells with the IVp-I-ON genotype increased over time after gut colonization, thus indirectly indicating an increase in the population with the ON/ON genotype. However, as shown in Fig 9C, colonization ability to intact germ-free mice intestine was similar between wild and OFF/ON mutant *B*. *fragilis* strains, These *in vivo* observation again supports the idea that the IVp-I/IVp-II-regulated OMV phenotype is an adaptation to membrane stress encountered in the special setting.

How do these OMVs protect *B*. *fragilis* cells from membrane attack in the presence of bile or human defensins? As shown in S4 Fig, exogenously added purified OMVs from ON/ON cells protected OFF/ON and OFF/OFF mutant cells from bile stress dose-dependently. This finding suggests that OMVs act as decoys, making it more difficult for bile or defensins to access *B*. *fragilis* cells. A recent proteomic analysis of *B*. *fragilis* OMVs showed that the vesicles of this species are enriched with acidic proteins and that the OMV protein profile was markedly different from that of the outer membrane [24]. This finding indicates the presence of a selective sorting system that directs certain proteins to OMVs. Many of the acidic proteins were glycosidases or proteases, thus suggesting a role for *B*. *fragilis* OMVs in nutrient uptake. Curiously, the OMV-enriched proteome did not contain the IVp-II related proteins (BF3397-BF3403). Comparative analysis of the protein profile in ON/ON mutant-derived OMVs with that in previously reported *B*. *fragilis* OMVs [24] is expected to determine the role of IVp-I/IVp-II-regulated hypervesiculating phenotype.

In conclusion, the present study provides the first identification of a genetic locus that is involved in *B*. *fragilis* OMV formation. In addition, we demonstrated that *B*. *fragilis* OMV formation is uniquely regulated by phase-variable DNA inversions. In a variety of Gram-negative pathogens, OMVs have been recognized as an important vehicle for virulence factors or signaling molecules. Despite the recent attention to OMV biology, the molecular basis of the OMV production machinery and the specific sorting system for enriched proteins in OMVs remain elusive. Although IVp-I/IVp-II-mediated vesicle formation might be only one mechanism of OMV production in *B*. *fragilis*, localization and protein-protein interaction analyses of IVp-II-regulated membrane proteins or IVp-I-controlled EPS may elucidate the precise mechanism of OMV production. Furthermore, tracing the fate of *B*. *fragilis* OMVs or the OMV contents (enriched proteins) *in vivo* may be facilitated by fluorescently labeling OMV-enriched proteins in the hypervesiculating ON/ON mutant. The data presented herein may enhance OMV research in this gut symbiont and provide new insights into human-microbe interactions via OMVs.

## Materials and Methods

### Bacterial strains and plasmids

The bacterial strains and plasmids used in the present study are listed in S3 Table. *Escherichia coli* DH5α was used as a host strain for plasmid construction. The *E*. *coli*-*Bacteroides* shuttle plasmids pVAL-1 [39] and pLYL05 [40] were used for genetic manipulations in *B*. *fragilis*. A suicide vector, pKK100 [18], was used to disrupt *B*. *fragilis* genes. *E*. *coli* strains were cultured in lysogeny broth (LB) or on LB agar plates. *B*. *fragilis* strains were anaerobically grown in liquid Gifu Anaerobic Medium (GAM; Nissui Pharmaceutical Co., Tokyo, Japan) or on GAM agar plates using the AnaeroPack System (Mitsubishi Gas Chemical Co., Inc., Tokyo, Japan) or in an anaerobic chamber conditioned with mixed gases (N_2_, 80%; CO_2_, 10%; and H_2_, 10%). When necessary, antibiotics were added to the media at the following concentrations: ampicillin (Amp), 50 μg/ml; cefoxitin (Cfx), 50μg/ml; erythromycin (Em), 10 μg/ml; and tetracycline (Tc), 10 μg/ml. To compare the cell sedimentation rate among BF2766 *B*. *fragilis* mutants, overnight cultures were statically maintained at room temperature in an anaerobic chamber. In competitive growth assay, equivalent number (3.6 x 10^9^ colony-forming unit/ml each) of wild and ON/ON mutant *B*. *fragilis* YCH46 cell was mixed, and the mixture was added to 1% into 10 ml of GAM broth. A part of the culture was periodically sampled and appropriate dilutions were plated onto GAM agar plates. Colony PCR was performed on at least 96 colonies per sample with primer pair encompassing the deletion site in BF2766 to determine the ratio of wild type and ON/ON mutant cells.

### Gene disruption and site-directed mutagenesis of *B*. *fragilis*

The target genes of *B*. *fragilis* YCH46 were disrupted as previously described [18]. First, 2-kb DNA fragments downstream and upstream of the target region were separately amplified and fused using a second PCR reaction via overlapping sequences that were incorporated into the primer sequences. The fused DNA fragments were then cloned into pKK100. The purified plasmid was subsequently introduced into *B*. *fragilis* by electroporation as previously described [41, 42], employing conditions of 2.5 kV, 25 μF, and 200 Ω. Em-resistant colonies, in which the target plasmid had integrated into the chromosome through a single genetic crossover, were selected and grown in non-selective GAM broth. Dilutions of the overnight culture were spread onto GAM agar plates to provide an appropriate colony number; the plates were then replica plated onto GAM and GAM/Em plates to screen for Em-sensitive clones that resolved the diploid through a second homologous recombination. The expected mutants and revertants were discriminated by PCR using primers flanking the deletion site. In the case of *B*. *fragilis* NCTC9343, the fusion PCR products were first cloned into pLYL05 and subsequently introduced into the strain by electroporation to modify the plasmid by using a host cell restriction/modification system. After the plasmid was purified from *B*. *fragilis* NCTC9343, the *Xba*I fragment containing a replication protein gene for *Bacteroides* was removed by restriction digestion. The remaining fragment (which could not replicate in *Bacteroides*) was re-ligated and electroporated into *B*. *fragilis* NCTC9343 to integrate it into the target site. Cfx-resistant colonies were selected and replica plated to obtain the expected deletion mutants as described for strain YCH46. Gene replacement for site-detected mutagenesis was performed according to the process described above. The primer sequences used for gene disruption and replacement are listed in S4 Table. Synthetic oligonucleotide primers were purchased from Sigma-Aldrich Japan Co., Ltd. (Tokyo, Japan). DNA sequencing was performed on an ABI PRISM 3100 Genetic Analyzer (Applied Biosystems) by using the ABI PRISM BigDye Terminator Cycle Sequencing Ready Reaction Kit, version 1.1 (Applied Biosystems).

### Promoter inversion assay

The orientations of the IVp-I and IVp-II promoters relative to their downstream genes were assessed by PCR as previously described [22]. As shown in Fig 1C, the PCR primers F and R were designed outside of the IRs and primer M was designed from the region between the IRs. Two amplification reactions were set up for each promoter region: one was conducted with primers F and M (to detect promoters in the ON orientation) and the other was conducted with R and M (to detect promoters in the OFF orientation). PCR was performed under the following conditions: preheating at 95°C for 1 min, followed by 35 cycles of denaturation at 95°C for 30 s, annealing at 55°C for 30 s, and extension at 72°C for 1 min, with a final extension at 72°C for 5 min. The PCR products were visualized by ethidium bromide staining after electrophoresis on a 2% agarose gel. To examine the bile stress response, quantitative real-time PCR analyses were performed using genomic DNA to compare the ON/OFF ratios of the IVp-I or IVp-II promoters. Briefly, *B*. *fragilis* cells were grown anaerobically in GAM broth with 0%, 1% or 5% bile (Wako Chemical Co. Ltd., Tokyo) at 37°C for 8 h. The cells were harvested, and genomic DNA was extracted using the Easy-DNA Kit (Invitrogen). Quantitative PCR analyses were performed using SYBR Premix Ex Taq II (Takara) under the following conditions: preheating at 95°C for 30 s, followed by 40 cycles of denaturation at 95°C for 15 s, annealing at 55°C for 30 s, and extension at 60°C for 34 s in an ABI PRISM 7500 (Applied Biosystems). Genomic DNA from the BF2766 mutant (IVp-I/IVp-II ON/ON genotype) was used as the quantification standard. The *rpoD* gene was amplified to normalize the individual DNA samples. The oligonucleotide sequences of the primers used for each target gene are listed in S4 Table.

### RNA isolation and quantitative real-time PCR

Total RNA was extracted from mid-logarithmic-phase cultures (OD_660_; 0.4–0.6) of *B*. *fragilis* strains using the hot-phenol method [43]. The RNA was further purified using the RNeasy CleanUp Kit (Qiagen) and was treated with TURBO DNA-*free* (Ambion) to remove contaminating DNA. The total RNA was reverse transcribed using a PrimeScript RT reagent Kit (Takara Shuzo Co., Ltd., Otsu, Japan) with random hexamers at 37°C for 15 min. Reverse transcription was terminated by heating the mixtures at 85°C for 5 sec. The cDNA products were subsequently amplified using SYBR Premix Ex Taq II (Takara) under the following conditions: preheating at 95°C for 10 s, followed by 40 cycles of 95°C for 5 s and 60°C for 34 s in an ABI PRISM 7500 (Applied Biosystems). All samples were run in triplicate. Threshold cycle values were normalized to the levels of *rpoD* transcripts, and changes were calculated using the 2^-∆∆CT^ method [44]. The oligonucleotide sequences of the primers used for each target gene are listed in S4 Table.

### Electron microscopy

Cultures from the four *B*. *fragilis* IVp-I/IVp-II promoter genotypes with BF2766 deletions were fixed overnight with 2% glutaraldehyde in PBS at 4°C. For scanning electron microscopy (SEM), each cell sample was dehydrated with a series of ethanol solutions ranging in 10% increments from 50% (vol/vol) ethanol in distilled water to absolute ethanol. All samples were dried to the critical point using a critical point dryer, coated with gold, and examined by SEM (Hitachi S-800; Hitachi, Tokyo, Japan). For transmission electron microscopy (TEM), the fixed cells were pelleted by centrifugation at 3,000 x *g* for 10 min. Each sample of cells was fixed overnight in 1% osmic tetroxide. The specimens were dehydrated in acetone and embedded in epoxy resin (EMbed 812). The embedded materials were sectioned with a diamond knife on an LKB ultramicrotome and were stained with uranyl acetate and lead citrate. The stained sections were mounted on copper grids and observed in a Hitachi electron microscope H-700 at 75 kV.

### DNA microarray

The *B*. *fragilis* YCH46 DNA microarray from NimbleGen Systems, which includes 4,527 target genes with at least 8 unique 60-mer synthetic oligonucleotide probes for each gene, was used for the comparative transcriptomic analysis of the mutant strains. The cDNA synthesis, hybridization, and scanning were performed at NimbleGen. The microarray data were analyzed by quantile normalization and robust multiarray averaging [45]. The normalized data were processed with ArrayStar software (DNASTAR). The samples were filtered to identify differential expression that showed >4-fold induction in the ON/ON (IVp-I/IVp-II promoters) genetic background. Student’s *t* test was used for the analyzing the mean log ratios of two samples, and Bonferroni’s adjustment for multiple testing was applied, as rigorous criteria for significant changes in signal intensity. Changes with a *p*-value of less than 0.05 (*p* < 0.05) were considered statistically significant.

### Quantification of OMVs

The amount of OMVs produced by *B*. *fragilis* cells was monitored based on the protein concentrations in the culture supernatant as previously reported [46]. Briefly, *B*. *fragilis* cells were anaerobically cultured in 10 ml of GAM broth at 37°C for 12 h. In case of large scale OMVs purification, cells were cultured in 50–1,000 ml of defined minimal medium (DMM) [47] at 37°C for 24 h. The cultures were subsequently centrifuged at 6,000 x *g* for 10 min at 4°C, and the supernatants were filtered through 0.45-μm pore-size filters. The filtrate was subjected to ultracentrifugation at 150,000 x *g* for 3 h at 4°C. The pellets were washed three times with phosphate-buffered saline (PBS) and resuspended in 200 μl of PBS. Protein concentrations were measured using the Bradford method [48].

### FLAG tag fusion and Western blotting

To evaluate the effect of IVp-II promoter orientation on the expression of the downstream gene (BF3397), the amount of intracellular FLAG-tagged BF3397-encoded protein was monitored. First, BF3397 was fused to a 3 x FLAG tag-encoding sequence at the 3’ end by using PCR. The obtained product was cloned into pKK100. The constructed plasmid was integrated into the *B*. *fragilis* chromosome, and the original BF3397 gene was replaced with the BF3397-3 x FLAG tag fusion gene by homologous recombination. Immunoprecipitation and Western blotting were performed on the *B*. *fragilis* cells with or without exposure to 5% bile (Wako Chemical Co. Ltd., Tokyo) in GAM broth. The cells were harvested following 12-h of incubation in media and were subsequently resuspended in PBS supplemented with an EDTA-free complete protease inhibitor cocktail (Roche). After disrupting the cells by sonication and subsequent centrifugation (13,000 x *g* for 20 min), the supernatants (S13) were used for immunoprecipitation. One microgram of mouse anti-FLAG M2 antibody (Sigma) was added to S13 (70 μg protein), and the mixtures were incubated with rotation for 12 h at 4°C. After the addition of Dynabeads protein G (25 μl; Invitrogen), the mixture was further incubated for 2 h. The Dynabeads were washed 4 times in PBS and eluted by boiling with SDS-PAGE loading buffer for 5 min. Aliquots (20 μl) of the samples were loaded onto 10% SDS-PAGE gels, and the separated protein bands were transferred to a PVDF membrane using a Mini Trans-blot cell (Bio-Rad). The membrane was blocked with SuperBlock Blocking Buffer (Thermo) overnight at 4°C, reacted with mouse anti-FLAG M2 antibody for 30 min at room temperature, and subsequently incubated with horseradish peroxidase-linked secondary antibody (anti-mouse IgG) for 1 h at room temperature. Antibody binding was visualized with ECL Plus detection reagent (GE) and X-ray film.

### Preparation of outer membrane protein

Outer membrane protein purification was performed as follows. The overnight culture (50 ml) of FLAG-tagged strain (TSRM2766ON/ON BF3397::FLAG) in DMM broth were pelleted (6,000 x *g* for 10 min), and resuspended in 50 mM Tris-HCl (pH8.0), 150 mM NaCl, 50 mM MgCl_2_ containing EDTA-free complete protease inhibitor cocktail (Roche). After disrupting the cells by sonication, cell debris was removed by centrifugation at 6,000 x *g* for 10 min at 4°C, and the supernatant was further fractionated to cytoplasmic fraction (supernatant) and membrane fraction (pellet) by ultracentrifugation at 100,000 x *g* for 1 h at 4°C. The pellet was washed twice with 50 mM Tris-HCl (pH8.0), 150 mM NaCl, 50 mM MgCl_2_ containing EDTA-free complete protease inhibitor cocktail, resuspended in the same buffer containing 1.5% (v/v) Triton X-100 and then incubated at 25°C for 1 h. The outer membrane fractions were recovered by centrifugation at 100,000 x *g* for 1 h at 4°C.

### Assessment of resistance to bile and human antimicrobial peptides

*B*. *fragilis* cells were anaerobically grown in GAM broth overnight at 37°C. An aliquot of culture (30 μl) was inoculated into 3 ml of fresh GAM broth and cultured until late-log phase (OD_600_:1.3–1.6). After harvesting and washing with PBS, the cells were resuspended in PBS and subsequently treated with 5% bile (Wako), 0.5 μM LL-37 (Anaspec), or human defensins (10 μM α-defensin 5, 10 μM β-defensin 1, 10 μM β-defensin 2, or 0.5 μM β-defensin 3; all purchased from Peptide Institute, Japan). After the suspensions were incubated at 37°C, the viable cells numbers were measured periodically by plating 0.1 ml of the suspensions on GAM agar plates. The survival rate was calculated from the ratios of viable cells in the suspensions containing bile, LL-37, or human defensins to those in the suspensions without antimicrobial substances.

### Protection assay with OMVs

We determined whether the purified OMVs from the ON/ON mutant could protect the non-vesiculating cells (OFF/ON or OFF/OFF mutants) from bile stress. In briefly, the ON/ON mutant-derived OMVs (10 or 50 μg/ml of final concentration) or PBS was added to non-vesiculating cell suspensions. After the suspensions were treated with 5% bile at 37°C for 30 min, the viable cells counts were measured periodically by standard plating assay with GAM agar plates. The survival rate was calculated from the viable cell counts in samples with/without exposure to 5% bile.

### Intestinal colonization assay in germ-free mice

Three male germ-free BALB/cA mice (8 weeks old, Clea Japan Inc.) were orally inoculated with a suspension of wild-type *B*. *fragilis* YCH46 (7.9 x 10^7^ colony-forming units). Fecal samples were collected 3, 7, 10, and 14 days after inoculation. DNA was purified from the inoculum and from the fecal samples. ON/OFF ratio with respect to IVp-I or IVp-II in each sample was determined by qPCR.

Competitive colonization assay of wild type and OFF/ON mutant *B*. *fragilis* YCH46 was performed by orally inoculating the mixture of equivalent number of each type of cells (2.3 x 10^8^ colony-forming units) to five male germ-free BALB/cA mice (8 weeks old, Clea Japan). Fecal samples were collected at 3, 7, 10, and 14 days after inoculation, and appropriate dilutions of the samples were prepared and spread on GAM agar plates. Colony PCR was performed on at least 96 colonies per sample with primer pair encompassing the deletion site in BF2766 to compare population levels of mutants with wild type cells in the mouse intestine. In this experiment, mice were kept in a vinyl isolator to maintain their gnotobiotic condition.

### Microarray accession numbers

The microarray data have been deposited in the Gene Expression Omnibus database (www.ncbi.hlm.nih.gov/projects/geo) under the following accession numbers: normalized data, GSE69071; platform, GPL13213; and raw data files, GSM1692522 to GSM1692525.

### Ethics

Animal experiment was carried out in accordance with Japanese legislation (Act on Welfare and Management of Animals, 1973, revised in 2012) and guidelines under the jurisdiction of the Ministry of Education, Culture, Sports, Science and Technology, Japan (Fundamental Guidelines for Proper Conduct of Animal Experiment and Related Activities in Academic Research Institutions, 2006). The protocols of animal experiments were approved by the committee of animal experiment of the Tokushima University (Assurance Number; 08181). Animal care, housing, feeding, sampling, observation, and environmental enrichment were performed in accordance with the guidelines.

## Supporting Information

S1 Fig **Schematic map of the IVp-I region in ON/ON mutant of YCH46 (A) and NCTC9343 (B).** IR indicates the inverted repeat sequence. IRs (indicated as IR2) are shown by bold letters.
(TIFF)

S2 Fig SEM observation of IVp-I/IVp-II-locked *B*. *fragilis* mutants. (A) SEM observation of the ON/ON mutants of YCH46 (upper panel) and NCTC9343 (lower panel) at high magnification (x 30,000). The ON/ON mutant of NCTC9343 was constructed after disrupting the BF2694 gene, reported as *tsr19*. (B) SEM observation of the four combinations of NCTC9343 locked mutants with respect to IVp-I/IVp-II orientation (x 13,000). Only the ON/ON mutant showed the hypervesiculation phenotype similar to YCH46.
(TIF)

S3 Fig EPS production is necessary for OMV formation in *B*. *fragilis*. BF2769, which encodes a tyrosine protein kinase, was deleted from the ON/ON mutant. BF2771, which is located outside the EPS locus and is independent of IVp-I, was also deleted. SEM examination was performed on these mutants. The BF2769 deletion abrogated the hypervesiculation phenotype, whereas the BF2771 deletion had no effect on OMV formation. Plasmid complementation of BF2769 restored hypervesiculation. The bars indicate 1 μm.
(TIF)

S4 Fig Protection of non-vesiculating cells from the bile stress by exogenously added OMVs. The OMVs purified from ON/ON cell culture (final concentration of 10 μg/ml or 50 μg/ml) or PBS (pH7.4) were added to the mid-log-phase OFF/ON and OFF/OFF mutant cell suspensions. These suspensions were treated with 5% bile. After 30-min incubation at 37°C, the surviving *B*. *fragilis* cells were enumerated by standard plate culture. The columns show the ratios of surviving cells to the initial viable cell counts. The data are expressed as the mean ± standard deviation. The differences were statistically analyzed by ANOVA, followed by Tukey’s test. The *p*-values of less than 0.01 are indicated by asterisks (*).
(TIF)

S1 Table Summary of the genes in the regions downstream of IVp-I and IVp-II. (DOC)

S2 Table Genes with expression levels induced >4-fold in the ON/ON mutant. (DOC)

S3 Table Bacterial strains and plasmids used in the present study. Abbreviations: Ap, ampicillin; Cfx, cefoxitin; Em, erythromycin; Tc, tetracycline.
(DOC)

S4 Table PCR primers used in the present study. ^*a*^ Restriction sites (underlined) and overlapping sequences (boldcase) for fusion PCR that were incorporated into the primer sequences.
(DOC)
