# Supplementary figures and images for: DNA Inversion Regulates Outer Membrane Vesicle Production in Bacteroides fragilis
Source: PLoS One. 2016 Feb 9;11(2):e0148887. doi: 10.1371/journal.pone.0148887 (PMC4747536; doi:10.1371/journal.pone.0148887)

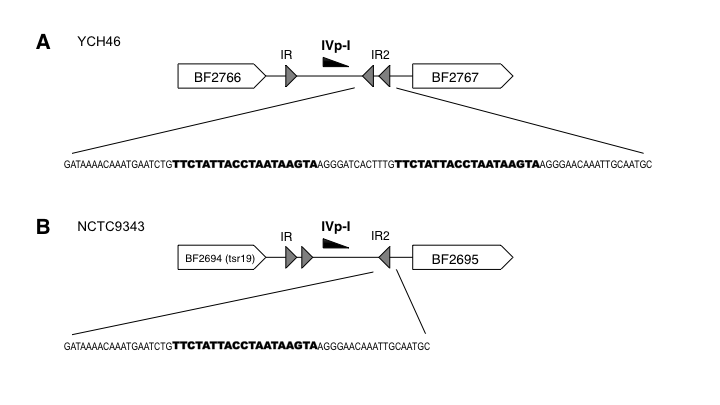

Supplement: S1 Fig — Schematic map of the IVp-I region in ON/ON mutant of YCH46 (A) and NCTC9343 (B). IR indicates the inverted repeat sequence. IRs (indicated as IR2) are shown by bold letters. (TIFF) [file pone.0148887.s001.tiff]

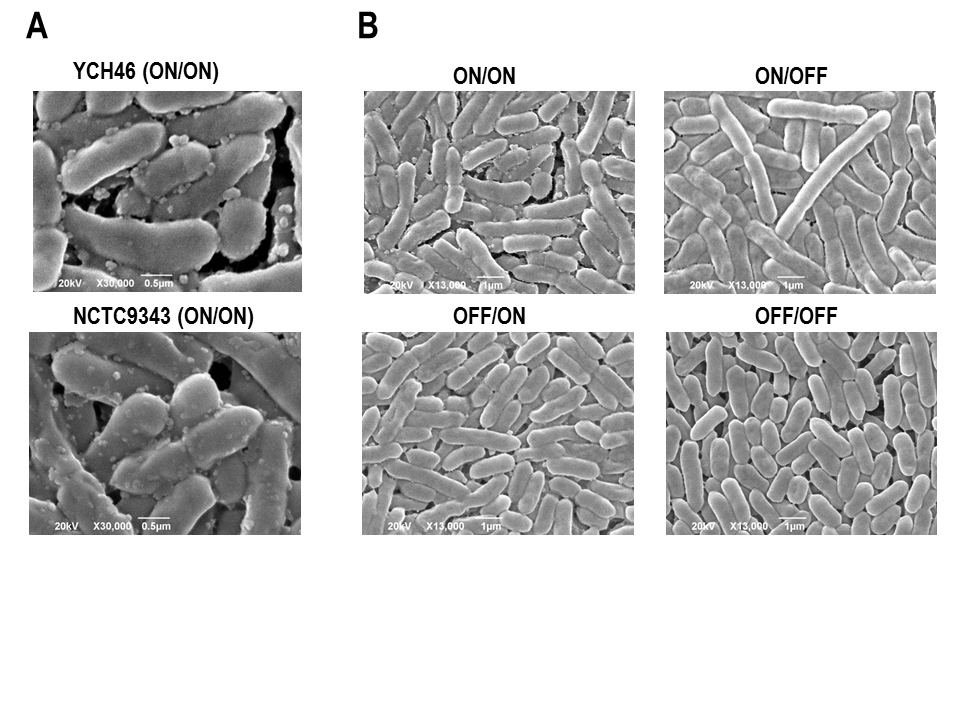

Supplement: S2 Fig — (A) SEM observation of the ON/ON mutants of YCH46 (upper panel) and NCTC9343 (lower panel) at high magnification (x 30,000). The ON/ON mutant of NCTC9343 was constructed after disrupting the BF2694 gene, reported as tsr19. (B) SEM observation of the four combinations of NCTC9343 locked mutants with respect to IVp-I/IVp-II orientation (x 13,000). Only the ON/ON mutant showed the hypervesiculation phenotype similar to YCH46. (TIF) [file pone.0148887.s002.tif]

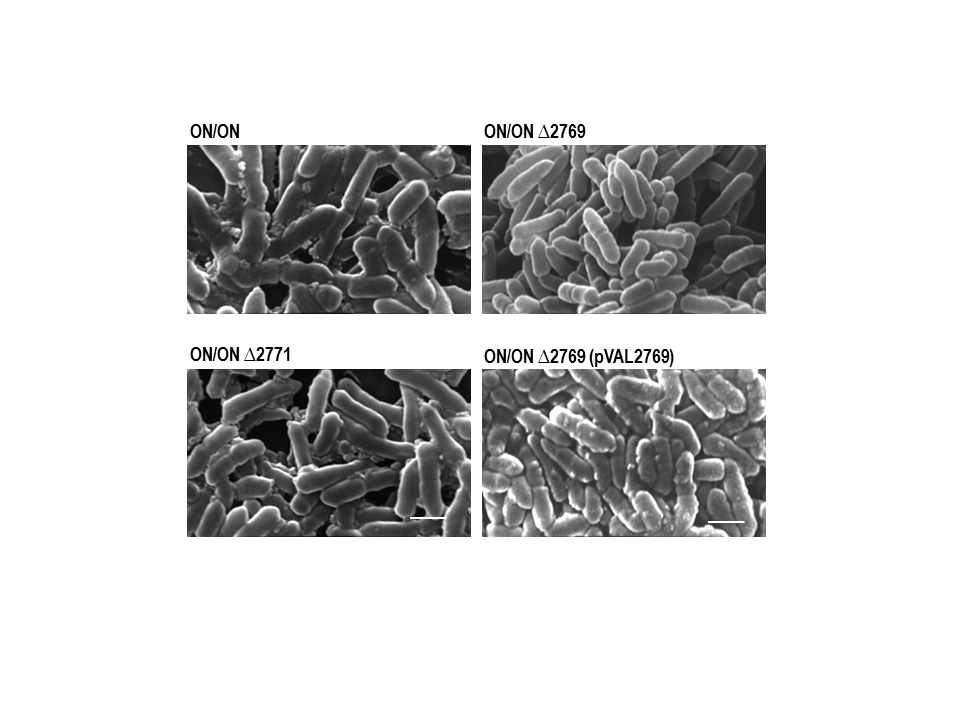

Supplement: S3 Fig — BF2769, which encodes a tyrosine protein kinase, was deleted from the ON/ON mutant. BF2771, which is located outside the EPS locus and is independent of IVp-I, was also deleted. SEM examination was performed on these mutants. The BF2769 deletion abrogated the hypervesiculation phenotype, whereas the BF2771 deletion had no effect on OMV formation. Plasmid complementation of BF2769 restored hypervesiculation. The bars indicate 1 μm. (TIF) [file pone.0148887.s003.tif]

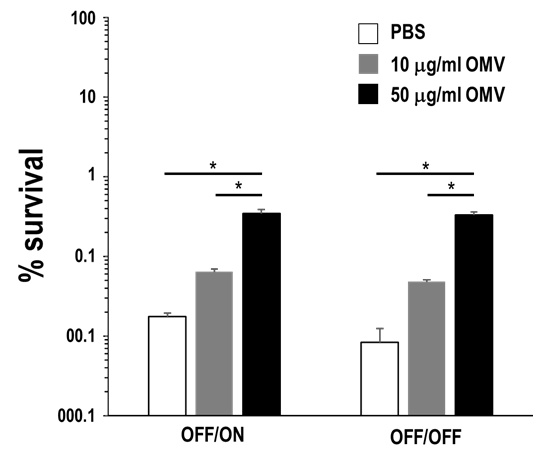

Supplement: S4 Fig — The OMVs purified from ON/ON cell culture (final concentration of 10 μg/ml or 50 μg/ml) or PBS (pH7.4) were added to the mid-log-phase OFF/ON and OFF/OFF mutant cell suspensions. These suspensions were treated with 5% bile. After 30-min incubation at 37°C, the surviving B. fragilis cells were enumerated by standard plate culture. The columns show the ratios of surviving cells to the initial viable cell counts. The data are expressed as the mean ± standard deviation. The differences were statistically analyzed by ANOVA, followed by Tukey’s test. The p-values of less than 0.01 are indicated by asterisks (*). (TIF) [file pone.0148887.s004.tif]
