# Supplementary material for: DNA Inversion Regulates Outer Membrane Vesicle Production in Bacteroides fragilis
Source: PLoS One. 2016 Feb 9;11(2):e0148887. doi: 10.1371/journal.pone.0148887 (PMC4747536; doi:10.1371/journal.pone.0148887)
Supplement: S1 Table — (DOC) [file pone.0148887.s005.doc]

**Table S1. Summary of genes on the downstream region of IVp-I and IVp-II.**

| Genes | Amino acids (a.a.) | Difinition | Motif | Paralog | Ortholog |
| --- | --- | --- | --- | --- | --- |
| IVp-I region |  |  |  |  |  |
| BF2767 | 467 | Glycosytransferase | CoA_binding_3 (CoA-binding domain)  Bac_transf (Bacterial sugar transferase) | non | BF2695 (NCTC9343)  BVU_2676  BT_0480  BDI_0654 |
| BF2768 | 262 | Polysaccharide export outer membrane protein | SLBB (SLBB domain)  Poly_export (Polysaccharide biosynthesis/export protein) | non | BF2696 (NCTC9343)  BVU_2675  BT_0398  BDI_0425 |
| BF2769 | 801 | Tyrosine-protein kinase | Wzz (Chain length determinant protein)  GNVR (G-rich domain on putative tyrosine kinase)  AAA_31 (AAA domain)  CbiA (CobQ/CobB/MinD/ParA nucleotide binding domain) | non | BF2697 (NCTC9343)  BVU_2674  BT_0482  BDI_0125 |
|  |  |  |  |  |  |
| IVp-II region |  |  |  |  |  |
| BF3397 | 399 | Hypothetical protein (membrane protein) | Mfa2 (Fimbrillin-A associated anchor proteins Mfa1 and Mfa2) | BF1563 | BF3138 (NCTC9343) |
| BF3398 | 378 | Hypothetical protein (soluble protein) | non | non | BF3139 (NCTC9343) |
| BF3399 | 377 | Hypothetical protein (soluble protein) | non | non | BF3140 (NCTC9343) |
| BF3400 | 369 | Hypothetical protein (soluble protein) | non | non | BF3141 (NCTC9343) |
| BF3401 | 310 | Hypothetical protein (lipoprotein) | non | BF1562  BF2211 | BF3142 (NCTC9343) |
| BF3402 | 678 | Hypothetical protein (membrane protein) | DUF4480 (Domain of unknown function)  OmpA family  CarboxypepD_reg (Carboxypeptidase regulatory-like domain) | BF1764  BF4485  BF2125  BF1609  BF1783 | BF3143 (NCTC9343) |
| BF3403 | 180 | Hypothetical protein (membrane protein) | DUF3575 (Protein of unknown function) | BF3229  BF1481 | BF3144 (NCTC9343)  BDI_3526  BT_1451  PGN_0178  BVU_2683 |
