# Supplementary material for: DNA Inversion Regulates Outer Membrane Vesicle Production in Bacteroides fragilis
Source: PLoS One. 2016 Feb 9;11(2):e0148887. doi: 10.1371/journal.pone.0148887 (PMC4747536; doi:10.1371/journal.pone.0148887)
Supplement: S2 Table — (DOC) [file pone.0148887.s006.doc]

**Table S2. The genes whose expressions were induced >4-fold in the only ON/ON mutant.**

| Gene | Function | Fold change  (OFFON/ONON) | Fold change  (OFFOFF/ONON) | Fold change  (ONOFF/ONON) |
| --- | --- | --- | --- | --- |
| BF3397 | hypothetical protein | 29.1 down | 279 down | 256 down |
| BF3398 | hypothetical protein | 47.8 down | 166 down | 186 down |
| BF3399 | hypothetical protein | 62.5 down | 127 down | 129 down |
| BF3400 | hypothetical protein | 89.7 down | 180 down | 179 down |
| BF3401 | hypothetical protein | 148 down | 176 down | 198 down |
| BF3402 | hypothetical protein | 130 down | 213 down | 247 down |
| BF3403 | hypothetical protein | 42.9 down | 62.3 down | 55.1 down |
| BF3406 | hypothetical protein | 12.2 down | 15.2 down | 17.8 down |
| BF3407 | RNA polymerase ECF-type sigma factor | 15.3 down | 21.6 down | 15.4 down |
| BF3410 | hypothetical protein | 14.2 down | 15.0 down | 20.1 down |
| BF4531 | hypothetical protein | 5.23 down | 6.48 down | 4.55 down |
