# Supplementary material for: DNA Inversion Regulates Outer Membrane Vesicle Production in Bacteroides fragilis
Source: PLoS One. 2016 Feb 9;11(2):e0148887. doi: 10.1371/journal.pone.0148887 (PMC4747536; doi:10.1371/journal.pone.0148887)
Supplement: S3 Table — Abbreviations: Ap, ampicillin; Cfx, cefoxitin; Em, erythromycin; Tc, tetracycline. (DOC) [file pone.0148887.s007.doc]

| **Table S3. Bacterial strains and plasmids used in this study.** | |  |
| --- | --- | --- |
| Strain or plasmid | Relevant genotype or description | Reference or source |
| Strains |  |  |
| *Escherichia coli* |  |  |
| DH5α | F^－^ Φ80d*lacZ*ΔM15 Δ(*lacZYA-argF*)U169 *deoR* *recA*1 *endA*1 *hsdR*17(rK^－^, mK^＋^) *phoA* *supE*44 λ^－^ *thi*-1 *gyrA*96 *relA*1 | Invitrogen |
| *Bacteroides fragilis* |  |  |
| YCH46 | Clinical isolate, parental strain for all deletion mutants | [6] |
| NCTC9343 | Type strain, appendix abscess | NCTC |
| TSRM2766ON/ON | Locked ON/ON mutant of IVp-I/II with deletion of BF2766 (YCH46) | This study |
| TSRM2766OFF/ON | Locked OFF/ON mutant of IVp-I/II with deletion of BF2766 (YCH46) | [18] |
| TSRM2766OFF/OFF | Locked OFF/OFF mutant of IVp-I/II with deletion of BF2766 (YCH46) | This study |
| TSRM2766ON/OFF | Locked ON/OFF mutant of IVp-I/II with deletion of BF2766 (YCH46) | This study |
| TSRM2694ON/ON | Locked ON/ON mutant of IVp-I/II with deletion of BF2694 (NCTC9343) | This study |
| TSRM2694OFF/ON | Locked OFF/ON mutant of IVp-I/II with deletion of BF2694 (NCTC9343) | This study |
| TSRM2694OFF/OFF | Locked OFF/OFF mutant of IVp-I/II with deletion of BF2694 (NCTC9343) | This study |
| TSRM2694ON/OFF | Locked ON/OFF mutant of IVp-I/II with deletion of BF2694 (NCTC9343) | This study |
| ON/ONΔ2769 | TSRM2766ON/ON mutant with deletion of BF2769 | This study |
| ON/ONΔ2771 | TSRM2766ON/ON mutant with deletion of BF2771 | This study |
| ON/ONΔ2767-2773 | TSRM2766ON/ON mutant with deletion of BF2767-BF2773 | This study |
| ON/ONΔ3403 | TSRM2766ON/ON mutant with deletion of BF3403 | This study |
| ON/ONΔ3402-3403 | TSRM2766ON/ON mutant with deletion of BF3402-BF3403 | This study |
| ON/ONΔ3397-3403 | TSRM2766ON/ON mutant with deletion of BF3397-BF3403 | This study |
| ON/ONΔIVp-I | TSRM2766ON/ON mutant with deletion of IVp-I | This study |
| ON/ON P1 SDM | TSRM2766ON/ON mutant with site-directed mutation of IVp-I | This study |
| ON/ONΔ3407 | TSRM2766ON/ON mutant with deletion of BF3407 | This study |
| BF3397::FLAG | YCH46 derivative expressing BF3397-3×FLAG | This study |
| Plasmids |  |  |
| pBluescript II KS | *E. coli* cloning vector; Ap^R^ | Stratagene |
| pLYL05 | *E. coli-Bacteroides* shutle vector; Ap^R^ in *E. coli*, Cfx^R^ in *Bacteroides* | [40] |
| pKK100 | Suicidal vector for *Bacteroides*; 3.8-kb Em/Tc resistance element from pE5-2 cloned into the *Xmn* I site of pBluescript II KS | [18] |
| pVAL-1 | *E. coli-Bacteroides* shutle vector; Ap^R^ Tc^R^ in *E. coli*, Em^R^ in *Bacteroides* | [39] |
| pVAL-Exp | *Bacteroides* expression vector, IS*1224*/*cepA* hybrid promoter [23], restriction sites (*Nde* I, *Not* I, *Xba* I and *Sal* I), and the transcription terminator of BF1719 cloned into the *Nhe* I/*Nru* I site of pVAL-1 | [18] |
| pVAL2766 | BF2766 amplified with primers BF2766-NdeI and BF2766-XbaI from BF strain YCH46 cloned into the *Nde* I/*Xba* I site of pVAL-Exp | This study |
| pVAL2769 | BF2769 amplified with primers BF2769-NdeI and BF2769-XbaI from BF strain YCH46 cloned into the *Nde* I/*Xba* I site of pVAL-Exp | This study |
| pVAL3403 | BF3403 amplified with primers BF3403-F and BF3403-R from BF strain YCH46 cloned into the blunted *Nde* I/*Sal* I site of pVAL-Exp | This study |
| pVAL3397-3403 | BF3397-BF3403 amplified with primers BF3397-F and BF3403-R from BF strain YCH46 cloned into the blunted *Nde* I/*Sal* I site of pVAL-Exp | This study |

Abbreviations: Ap, ampicillin; Cfx, cefoxitin; Em, erythromycin; Tc, tetracycline.
