# Supplementary material for: DNA Inversion Regulates Outer Membrane Vesicle Production in Bacteroides fragilis
Source: PLoS One. 2016 Feb 9;11(2):e0148887. doi: 10.1371/journal.pone.0148887 (PMC4747536; doi:10.1371/journal.pone.0148887)
Supplement: S4 Table — a Restriction sites (underlined) and overlapping sequences (boldcase) for fusion PCR that were incorporated into the primer sequences. (DOC) [file pone.0148887.s008.doc]

| **Table S4. PCR primers used in this study.** | |  |
| --- | --- | --- |
| Primer name | Sequence (5'→3') *^a^* | Usage |
| BF2769-1 | GCTCTAGAATATTGCACCGCCGTGTAGTAC | BF2769 knockout (upstream fragment) |
| BF2769-2 | **TAATAGCGGCGCAGATGATG**ACCGAGGCTACAAA | BF2769 knockout (upstream fragment) |
| BF2769-3 | **CATCATCTGCGCCGCTATTA**CGGATACGGAAAGA | BF2769 knockout (downstream fragment) |
| BF2769-4 | GCTCTAGAAAGTGGTGGATAAGGCTGTGGA | BF2769 knockout (downstream fragment) |
| BF2771-1 | GCTCTAGATGATCGTGAACCTGGATAGC | BF2771 knockout (upstream fragment) |
| BF2771-2 | **GGTAGTGGCGGATATTGTGG**ATGACCCGACAGCA | BF2771 knockout (upstream fragment) |
| BF2771-3 | **CCACAATATCCGCCACTACC**GAATACGTAACTTC | BF2771 knockout (downstream fragment) |
| BF2771-4 | GCTCTAGAAGCAGTGGTTGAGAAATACGCG | BF2771 knockout (downstream fragment) |
| BF3403-1 | GGGGTACCTCTTTCCGCCCAAACTCCACAT | BF3403 knockout (upstream fragment) |
| BF3403-2 | **TTCCCGCAGAGCCCAGGAGA**GGGCATTTGTTTTT | BF3403 knockout (upstream fragment) |
| BF3403-3 | **TCTCCTGGGCTCTGCGGGAA**TAGGATATGCATCT | BF3403 knockout (downstream fragment) |
| BF3403-4 | GGGGTACCATTGAGCGTGGATATCGGAGTG | BF3403 knockout (downstream fragment) |
| BF3397_3403-1 | GGGGTACCGCACGAATGTAAGCTACACCAC | BF3397-BF3403 knockout (upstream fragment) |
| BF3397_3403-2 | **TTCCCGCAGATCATTCAAGG**TTGTTGGTGCCGCT | BF3397-BF3403 knockout (upstream fragment) |
| BF3397_3403-3 | **CCTTGAATGATCTGCGGGAA**TAGGATATGCATCT | BF3397-BF3403 knockout (downstream fragment) |
| BF3397_3403-4 (BF3403-4) | GGGGTACCATTGAGCGTGGATATCGGAGTG | BF3397-BF3403 knockout (downstream fragment) |
| BF3402_3403-1 | GGGGTACCATTTGGATAGGATGTGGGAGTG | BF3402-BF3403 knockout (upstream fragment) |
| BF3402_3403-2 | **TTCCCGCAGATGACTCCCGA**AATGGGAATTGAGG | BF3402-BF3403 knockout (upstream fragment) |
| BF3402_3403-3 | **TCGGGAGTCATCTGCGGGAA**TAGGATATGCATCT | BF3402-BF3403 knockout (downstream fragment) |
| BF3402_3403-4 (BF3403-4) | GGGGTACCATTGAGCGTGGATATCGGAGTG | BF3402-BF3403 knockout (downstream fragment) |
| BF2767_2773-1 | GCTCTAGATTCTATTTCCGCCATCAGTCCG | BF2767-BF2773 knockout (upstream fragment) |
| BF2767_2773-2 | **CGGTAGACGGACTGTACTTT**TAGAAATGTTTGCG | BF2767-BF2773 knockout (upstream fragment) |
| BF2767_2773-3 | **AAAGTACAGTCCGTCTACCG**GTTCTTTTTAACAC | BF2767-BF2773 knockout (downstream fragment) |
| BF2767_2773-4 | GCTCTAGAGCAATAAGGGGTAGGTATAGCGCA | BF2767-BF2773 knockout (downstream fragment) |
| IVp-I-1 | gctctagaatggacctgaagcgttatgg | IVp-I knockout (upstream fragment) |
| IVp-I -2 | **TTGCAATTGCTATTAT**CACTCCTCCTCC | IVp-I knockout (upstream fragment) |
| IVp-I -3 | **ATAATAGCAATTGCAA**TGCACACAGCAAG | IVp-I knockout (downstream fragment) |
| IVp-I -4 | GCTCTAGAATAATTGGCCATGCGAACCG | IVp-I knockout (downstream fragment) |
| P1 SDM-1 (IVp-I-1) | gctctagaatggacctgaagcgttatgg | Site-directed mutagenesis of IVp-I (upstream fragment) |
| P1 SDM-2 | **TGGTTTAATAGAGATAATAA**TTGAAACTCC | Site-directed mutagenesis of IVp-I (upstream fragment) |
| P1 SDM-3 | **TTATTATCTCTATTAAACCA**GCGATAAAAC | Site-directed mutagenesis of IVp-I (downstream fragment) |
| P1 SDM-4 (IVp-I-4) | GCTCTAGAATAATTGGCCATGCGAACCG | Site-directed mutagenesis of IVp-I (downstream fragment) |
| BF3407-1 | GCTCTAGAAACGTCCCAAATGCGGACTA | BF3407 knockout (upstream fragment) |
| BF3407-2 | **AAAAAACATGTAGAATAAAG**GAACATGGAAC | BF3407 knockout (upstream fragment) |
| BF3407-3 | **CTTTATTCTACATGTTTTTT**GTCTCTTTTAATCC | BF3407 knockout (downstream fragment) |
| BF3407-4 | GCTCTAGATATCCTGCTTCCTGTCCTGA | BF3407 knockout (downstream fragment) |
| BF3397-FLAG-1 | GGGGTACCAGGGAGTATCAGCACTCTGTCT | BF3397-3×FLAG knockin (upstream fragment) |
| BF3397-FLAG-2 | **GAACTTCCAGTTCGACGTCT**ACATTCGTATTGTGA | BF3397-3×FLAG knockin (upstream fragment) |
| BF3397-FLAG-3 | **AGACGTCGAACTGGAAGTTC**TGTTCCAGGG | BF3397-3×FLAG knockin (FLAG fragment) |
| BF3397-FLAG-4 | **TAATGGATTTTTACTATTTA**TCGTCGTCATCT | BF3397-3×FLAG knockin (FLAG fragment) |
| BF3397-FLAG-5 | **TAAATAGTAAAAATCCATTA**GAATTTTTCTGCC | BF3397-3×FLAG knockin (downstream fragment) |
| BF3397-FLAG-6 | GGGGTACCCCGCAAACGTATATTGGTCACC | BF3397-3×FLAG knockin (downstream fragment) |
| BF2694nctc-1 | GCTCTAGAAATCCGGCTCCTGAGTAATCTC | NCTC9343 BF2694 knockout (upstream fragment) |
| BF2694nctc-2 | **GTATACCCAGTGTGTT**CAAGAACACGG | NCTC9343 BF2694 knockout (upstream fragment) |
| BF2694nctc-3 | **AACACACTGGGTATAC**AACAAACGCCTC | NCTC9343 BF2694 knockout (downstream fragment) |
| BF2694nctc-4 | GCTCTAGAATAATTGGCCATGCGAACCG | NCTC9343 BF2694 knockout (downstream fragment) |
| BF2766-NdeI | GGAATTCCATATGATTAATACACCACGAAA | BF2766 cloning into pVAL-Exp for complementation studies |
| BF2766-XbaI | GCTCTAGATCACTCCTCCTCCTCTTTCT | BF2766 cloning into pVAL-Exp for complementation studies |
| BF2769-NdeI | GGAATTCCATATGAAAGAAGACTTATACGACG | BF2769 cloning into pVAL-Exp for complementation studies |
| BF2769-XbaI | GCTCTAGACTAATTATGCTTTTGTCCGT | BF2769 cloning into pVAL-Exp for complementation studies |
| BF3397-F | ATGAAATCATTTAAAATCATGC | BF3397-BF3403 cloning into pVAL-Exp for complementation studies |
| BF3403-F | ATGAAAAAGAGAGCATTCTT | BF3403 cloning into pVAL-Exp for complementation studies |
| BF3403-R | CTATTTGAGTATATAGATCAGGCTG | BF3397-BF3403 cloning into pVAL-Exp for complementation studies |
| IVp-I-F | GGGTATACAACAAACGCCTC | IVp-I inversion assay |
| IVp-I-M | TACTCAAACAGGAGTTTACA | IVp-I inversion assay |
| IVp-I-R | CTCCATGCTTACGATAGGAC | IVp-I inversion assay |
| IVp-II-F | CTGAGTTTGCAGAGCTTCTG | IVp-II inversion assay |
| IVp-II-M | AGTTGAGATAATAGTCGCAT | IVp-II inversion assay |
| IVp-II-R | AGCACTCTGTCTGCTAATGG | IVp-II inversion assay |
| Real-InvR | ATAACTACCATCTTGCTGTG | IVp-I real-time inversion assay |
| mpi-PCR1-new | TTATCTCTATTTTTGCAGCG | IVp-I real-time inversion assay |
| Real-Gro-R2 | ACTGACAAAATGACTGATGA | IVp-II real-time inversion assay |
| Real-Gro-M2 | AGAATCATTTGCATAGCCAT | IVp-II real-time inversion assay |
| mpi-PCR3 | GGTGCTATAGAGCACTACCTGCTTC | BF2766 deletion checking |
| mpi-PCR8 | CGCACTGATCACTGAAACCG | BF2766 deletion checking |
| Real-BF2767-F | CAAATCGTATGGCGTGCCTTA | Real-time PCR |
| Real-BF2767-R | GTGAGCGCGCAGGAAAA | Real-time PCR |
| Real-BF2769-F | TCCCTGATCGGGCAATACA | Real-time PCR |
| Real-BF2769-R | TCCGAGGAGTTGCGTAGCA | Real-time PCR |
| Real-BF3397-F | CCTCATCATCGCAAACACTGA | Real-time PCR |
| Real-BF3397-R | CGGGAACTTTGATGGTTCGT | Real-time PCR |
| Real-BF3403-F | TTGACTTCCATACTTTCCCAAGAGA | Real-time PCR |
| Real-BF3403-R | CCCAGGTTTATGGTCGTGGTT | Real-time PCR |
| Real-groES-F | TTAACCCAAAACAGCGAGAACA | Real-time PCR |
| Real-groES-R | TGCTGGAACGGAACTTGAAGT | Real-time PCR |
| Real-rpoD-F | TGGGCGAATCATCGTTCAC | Real-time PCR |
| Real-rpoD-R | TGGACGCTCCTTTCGTTGA | Real-time PCR |
| *^a^* Restriction sites and overlapping sequencies for fusion PCR, which were incorporated into the primer sequences, are underlined and shown in boldcase, respectively | | |
